# Oral Health Knowledge and Related Factors among Pregnant Women Attending to a Primary Care Center in Spain

**DOI:** 10.3390/ijerph16245049

**Published:** 2019-12-11

**Authors:** Carmen Llena, Tasnim Nakdali, José Luís Sanz, Leopoldo Forner

**Affiliations:** Department of Stomatology, Universitat de València, 46010 Valencia, Spain

**Keywords:** pregnancy, oral health, knowledge on oral health, self-care

## Abstract

Our aim was to assess the knowledge of pregnant women in terms of oral health and prevention, correlating it with socio-sanitary and educational factors, as well as self-care and oral health state referred. A total of 139 women from a Health Department in Comunidad Valenciana (Valencia, Spain) participated in the study. They underwent an auto-administered survey which included socio-economic and educational factors, self-care in terms of oral hygiene, referred oral health state, and general knowledge on prevention and oral health. Chi-squared test (χ2 test) and ANOVA (*p* < 0.05) were performed for the analysis. Variables significantly associated with general knowledge were included in a logistic regression analysis. Variables which explained general knowledge in terms of oral health were Spanish nationality (Exp B = 3.59 *p* = 0.017), secondary/bachelor or equivalent level of education (Exp B = 0.23 *p* = 0.010), medium or high level of self-care (Exp B = 0.146 *p* = 0.024 and Exp B = 0.208 *p* = 0.046, respectively), and medium or high knowledge on prevention (Exp B = 0.092 *p* = 0.003 and Exp B = 0.280 *p* = 0.017, respectively). Level of education, nationality, self-care, and knowledge on prevention and oral health were the factors that determined a greater level of general knowledge on oral health from the pregnant women.

## 1. Introduction

In the last decade, the importance of oral health during pregnancy has attracted the attention of those responsible for the caring of pregnant women and infants (foundations, agencies, health care providers, etc.) [1]. The World Health Organization (WHO) [2] has recognized that oral health is part of the preventive measures in health care for pregnant women and infants.

Pregnancy is a stage of life for women in which numerous physiological and lifestyle changes occur [3]. These changes are, in great measure, responsible for the manifestations that are produced in the oral cavity during pregnancy. Therefore, all oral components, including both soft and hard tissues, can be affected during this period [4]. This fact demonstrates the need for an adequate oral care in pregnant women.

The most frequent changes produced in the oral cavity during pregnancy are pregnancy gingivitis, with a prevalence of 60%–75% [3,5,6]; xerostomy, between 15% and 18% [4], pregnancy epulis, with a prevalence of approximately 5% [7]; dental erosion, which occurs in 75%–80% of cases [5], and halitosis, which is referred in around 13% of cases [8,9]. Likewise, the changes in dietary habits can be associated with an incremented risk of developing caries lesions or a progression of those already present [5,10].

Maternal oral health during pregnancy has implications in the development of the baby and in its health. Thus, the presence of nontreated active periodontal pathology can be associated to a higher risk of preeclampsia, preterm infants, or low birth weight [6]. Inflammation of the placenta leads to a lower secretion of key growth factors for the fetus, among which are the fibroblast growth factor (FBF) and the brain-derived neutrophic factor (BDNF). Studies in human microbiome have determined that microorganisms present in the placenta proceed in a greater measure from the oral cavity compared with vaginal or enteric pathways. The existence of a high systemic inflammatory response in pregnant women with periodontitis has also been demonstrated. Moderate to severe periodontitis has been associated to higher levels of c-reactive protein (CRP) and prostaglandin E2 (PGE2), which are important risk factors for adverse results in the pregnancy [11]. All these issues are not frequently known by pregnant women, and even health personnel who attend them are unfamiliar with them, which results in a lack of consideration of the importance of a good oral health state for the adequate development of the baby.

Nowadays, it is known that in the oral cavity of newborns, microorganisms can be found very early, and that the number of oral bacteria grows gradually from the exposition to environmental microbial sources. With the eruption of the temporary dentition, the number and complexity of oral microbiota increases [12]. There are studies which support the theory that children acquire *S. mutans* from their mother [13], but the transmission can also be produced from other members of the family or caregivers as the child increases contact with them. The sooner the oral colonization by *S. mutans* is produced, the greater the risk of caries development. Some longitudinal studies have reported that children who acquired *S. mutans* before 2 years of age showed a greater caries experience in both temporary and permanent dentition in comparison with children who reported a later colonization [14]. This highlights the importance of self-care and good oral health among mothers.

An interesting longitudinal study carried out by Dzidic et al. [15] about the microbial composition of a sample of children followed from birth to 7 years of age, demonstrated that feeding in the first months of age, either with breastmilk or infant formula, influences the microbial composition in the oral cavity of the infant.

A literature review carried out by Abou et al. [16] highlights the importance and effectiveness of early oral health promotion, even before the birth of the child. Nurses and midwives are in a potentially excellent position to collaborate with oral health education, both for mothers and children. In a study executed by Lucey [17], it was confirmed that a program for oral health promotion, based in repeated rounds of anticipated orientation initiated during pregnancy, was successful for reducing the incidence of early childhood caries. Diverse studies confirm that oral health promotion during pregnancy improves oral health of mothers and children [18].

In Spain, the prevalence of caries in temporary dentition has maintained in levels around 32% in the last 25 years [19]. This high prevalence cannot be reduced if no actions or measures are planned for health education among pregnant women and preventive measures from birth. On the other hand, we cannot forget that caries in temporary dentition is the primary risk factor for caries development in permanent dentition [20].

Based on the objectives from the worldwide program “Alliance for a Cavity Free Future” (ACFF) [21], it was raised as a primary goal that children born in 2026 are caries-free. With data available at present, the implementation of community programs aimed at pregnant women and the interdisciplinary collaboration of different professionals is the only way to approximate to the said objective.

We established as our hypothesis that the level of knowledge about oral health and basic care, both self-care and care for the baby’s oral health, will be scarce and thus insufficient to maintain a good oral health state for their future children. The aim of this descriptive study was to assess the general knowledge about oral health care of pregnant women and relate it to socio-sanitary and educational factors, as well as self-care and oral health state referred.

## 2. Materials and Methods

This is a descriptive, cross-sectional study that was approved by the Ethics Committee for Human Investigations from Universitat de València, with the procedure reference H1536938110050. The investigation was carried out following the rules of the Declaration of Helsinki of 1975, revised in 2013.

The sample for the study consisted of pregnant women who attended the midwife’s office to follow up on their pregnancy of three Health Centers of the Department of Health General Hospital of Valencia (Valencia, Spain), from October 2018 to March 2019. All pregnant women have the same possibility of being attended, not only those with any oral or general health problem. The consultation daily, those who were assigned an odd number in the cited list were selected and offered to participate in the study. A sample size of 137 participants was calculated, for an expected proportion of women with medium or average knowledge about oral health of 65%, a confidence interval of 95%, and a maximum error of 8%.

Our inclusion criteria were women over 18, with a confirmed pregnancy status, understanding of the Spanish language, and acceptance of participating in the study by signing an informed consent.

A total of 140 pregnant women were offered to participate, and all of them accepted by means of an informed consent (100% participation rate). However, one of the participants did not fill in the questionnaire in its completeness and was excluded from the study, so the analysis was conducted on 139 completed surveys.

Data was collected by means of an auto-administered questionnaire for the study sample, which was given by the midwives when they attended consultation. The questionnaire used was validated for Peruvian women [22], and some modifications were made in order to adjust it to the study population’s characteristics. To do so, terminology was adjusted to the Spanish language and a pilot study was carried out, by giving the questionnaires to 10 Spanish pregnant women to assure for a correct comprehension of them.

The questionnaire was structured in five main blocks: general information (eight questions), oral health self-care (five questions), referred oral health state (three questions), oral health knowledge (which was subdivided into two parts, one for knowledge about basic preventive care (nine questions) and one for general knowledge about oral health (17 questions)), forming a total of 42 questions.

For the data analysis related to knowledge, 1 point was assigned for correct answers and 0 for the wrong answers. Then, the number of correct answers in each block was calculated, and the level of knowledge was grouped and categorized as low (<50% of correct answers), medium (50%–70% of correct answers), or high (>70% of correct answers). The questions in the self-care block were also codified according to the 1/0 point system. In the referred oral health state block, 1 point was assigned to good oral health state and 0 for bad oral health state. The codification used to group the different blocks is shown in Table 1.

The statistical analysis was carried out using the statistical package SPSS v25.00 (SPSS, Inc., Chicago, IL, USA). A descriptive analysis for each variable was made, associating explanatory variables (socio-sanitary and educational data, self-care and referred oral state) with answer variables (basic knowledge about preventive care of the infant’s mouth and general knowledge about oral health). The association between quantitative and qualitative variables was carried out using the ANOVA test, and qualitative variables were further analyzed between them using Chi-squared test, using a significance level of *p* < 0.05.

## 3. Results

### 3.1. General Data

The sample was formed by 139 women with a mean age of 31.42 ± 5.43. Of these women, 80.6% (112 pregnant women) were Spanish, and the remaining 19.4% (27 pregnant women) were from the following nationalities: Romanian, Moroccan, Bulgarian, Ecuadorian, Pakistani, French, Portuguese, Argentinian, Venezuelan, Chinese, Italian, Bolivian, Colombian, and Peruvian. Of these, nine (33.3%) had been living 5 years or less in Spain, and 56.8% (79 women) of the sample were doing paid work. Within the group of women workers, according to the first digit of the International Classification of Occupations of 2011 (isco-88) [23], 34 (44.15%) were technicians, scientific, and intellectual professionals, 8 (10.39%) were professional support technicians, 12 (15.59%) were employed in accounting, administration, or other office jobs, and 23 (29.87%) were catering, personal, protection, and sales service workers, 15.8% of pregnant women had undergone primary education, 57.6% secondary education, and 26.6% university degrees. For 56.8% of the sample, it was their first pregnancy, and the remaining 43.2% had had children previously. Within the group who had had children previously, 70% reported having one child, 18.34% two children, and the remaining 11.66% three children. Only 10.1% of the sample had assisted to a specific training program about oral health care. Table 2 summarizes the previously described data.

### 3.2. Oral Hygiene and Self-Care

Within the group, 79.9% of the women indicated that they brushed their teeth at least twice a day, and 56.8% indicated that they used fluoridated toothpaste. It is worth noticing that 23% (33 women) did not know if the toothpaste they used contained fluoride. Of the women, 59% (82 women) of the sample did not use dental floss or interdental brush, 61.2% of them indicated that they brushed their tongue, and 42.2% of the pregnant women (59 women) had been to the dentist less than a year ago. These data are summarized in Table 3.

### 3.3. Referred Oral Health State among Pregnant Women

Of the sample, 10.1% affirmed that they had never had caries, 32.4% referred having all carious lesions treated, 39.9% did not know if they had carious lesions or not, and 26.6% knew they had carious lesions. Of the women, 15.8% indicated that their gums bled spontaneously, 33.8% referred bleeding while brushing their teeth, 28.8% said that the bleeding appeared during pregnancy, and 21.6% of them had never experienced gingival bleeding. Of the sample, 73.4% referred no pain or oral infection in the last 10 months. Table 4 summarizes the referred oral health state among the participants.

### 3.4. Knowledge about Prevention in Oral Health

Of the pregnant women, 81.3% did not know about basic preventive measures that were proposed. Of the women, 71.9% knew that tooth brushing served for eliminating bacterial plaque, and the majority of the sample (85.6%) knew that fluoride can prevent the development of caries. A total of 53 women (38.1%) did not consider that cleaning their child’s mouth before teeth eruption was important. Moreover, 22 women (15.8%) did not think that it was important to clean the mouth and teeth of their child once they had erupted. Of the sample, 94.2% (131 women) knew that leaving the child sleeping with the feeding bottle with sugary liquids was not good for their oral health, and 84.9% of the women offered nightly nursing on demand after teeth eruption. Only 36% would brush their teeth with fluoridated toothpaste. Of the women, 83.5% knew that the prolonged use of the pacifier or sucking their finger can contribute to the development of malocclusions in their child. Table 5 summarizes the knowledge about prevention in oral health among pregnant women.

### 3.5. General Knowledge about Oral Health

Of the sample, 62.6% were not aware that the diet of the mother can affect to the teeth of children. Only 28 women (20.1%) knew that they should take their child to the dentist for the first time when his or her first tooth has erupted. Of the pregnant women, 63.3% did not know the risk of early childhood caries development in primary teeth of the babies. Only 16 pregnant women (11.5%) knew that the use of dental radiographs during pregnancy is not absolutely contraindicated. Of the sample, 83.5% thought that dental anesthesia is contraindicated during pregnancy. Moreover, 92.1% affirmed that bad dietary habits, tobacco, alcohol, and so forth can affect negatively in the health of the baby. However, only 38.8% of women were aware that a mother’s health problem can affect her child’s health. Of the pregnant women, 92.8% did not know that teeth begin their formation in the first weeks of gestation, and 39.6% of the women were not aware that the eruption of the first temporary tooth often happens around 6 months of age. Of the pregnant women, 74.8% did not know that the oral cleaning of their children should start from birth. Only 28.1% of the pregnant women (39 women) knew that fluoride reinforces their children’s teeth. These data are summarized in Table 6.

### 3.6. Level of Self-Care, Referred Oral Health State, and Knowledge of Pregnant Women

The different questions raised about the four aspects being evaluated (oral self-care and hygiene, referred oral health state, knowledge about prevention of the health of the baby’s mouth, and general knowledge about oral health) were grouped in three levels: high, medium, and low; according to the codification shown in Table 1. In terms of oral hygiene and self-care and referred oral health state, only 10.8% and 20.9%, respectively, had a high level of self-care and referred a good oral health state. Of the pregnant women, 23.7% presented a high level of knowledge about prevention in oral health, and none of the surveyed presented a high level of general knowledge about oral health (Table 7).

### 3.7. Association between the Different Variables of the Study

Regarding the age of the pregnant women, referred oral health state was significantly better in older women compared with the younger ones (*p* = 0.009). Likewise, level of general knowledge was also superior in older women (*p* = 0.029). However, the rest of parameters were not associated significatively with age, as shown in Table 8.

Type of jobs were assessed (housewives/paid jobs) depending on the nationality of the pregnant women (Spanish or foreign), finding a significantly superior number of women with paid jobs within the Spanish women (64.3%), versus the foreigners (18.5%) at *p* < 0.001. However, in terms of level of education, no differences were found; 84.8% of Spanish women and 81.4% of foreigners had secondary or university studies. 

Oral self-care among pregnant women was significantly superior in Spanish women (*p* = 0.031). A quasi significant association was found between the level of education and oral self-care, being the pregnant women with higher education the ones with better level of oral self-care as opposed to those with only primary education (*p* = 0.055). It can be affirmed that the lower the general knowledge in oral health, the lower the level of self-care among pregnant women (*p* = 0.037). Data is summarized in Table 9.

In terms of referred oral health state, women with a foreign nationality had a worse oral health state than Spanish women (*p* = 0.014). Those with primary education presented a worse health state (*p* = 0.030). It was observed that women with better general knowledge about oral health had a better referred oral health state with significant values (*p* = 0.012), as shown in Table 9.

It was found that pregnant women who assisted to talks about oral health promotion had a better level of knowledge about prevention in oral health compared with those who did not assist, with quasi significant values (*p* = 0.051). Pregnant women with a greater level of general knowledge about oral health also presented a better level of knowledge about prevention in oral health (*p* = 0.003), as described in Table 9.

The association between general knowledge about oral health and the rest of variables is presented in Table 9. Spanish women showed better levels of general knowledge about oral health compared with foreigners (*p* = 0.010). Women who did not have a paid job presented lower levels of knowledge about oral health compared with those who did (*p* = 0.004). Pregnant women with higher education showed a greater knowledge of oral health compared with those who only possessed primary education (*p* = 0.015). Women who were in their first pregnancy had better knowledge than those who had already had a child (*p* = 0.032). It should be noted that for 62.5% of Spanish woman, this was their first pregnancy, while 66.7% of foreigners had had previous pregnancies, finding a significant association (*p* = 0.006) between nationality and number of pregnancies.

A logistic regression analysis was performed, considering the variables significantly associated with the general knowledge about oral health of pregnant women (Table 10). It can be seen that the variables that explained the level of knowledge about oral health of pregnant women were nationality, level of education, level of self-care, and knowledge about prevention in oral health.

## 4. Discussion

To our knowledge, in Spain, only two investigations have been carried out which study the level of knowledge and self-care among pregnant women, and its relation with personal, socio-economic, and educational factors. The first of them took place in Murcia in 2011 [24], and the second one in Granada in 2018 [25]. However, at an international level, more than 20 studies which evaluate the same parameters have been found.

The predominant nationality in the three investigations carried out in Spain was Spanish (80.1% in the study from Murcia [24], 100% in the one from Granada [25], and 80.6% in this study). In terms of level of education, in the three studies, the highest percentage was found in secondary education.

For self-care, in the study carried out by Assery [26] (Saudi-Arabia, 2016), 33.3% of the sample brushed their teeth twice a day. In the study by Avula et al. [27] (India, 2013), 20.9% brushed their teeth twice a day. In the one by Gaffar et al. [28] (Saudi-Arabia, 2016), the percentage was 51%, 100% in Bamanikar and Kee’s study [29] (Brunei, 2013), and 84% in the one by Martínez-Beneyto et al. [24] (Murcia -Spain-, 2011). However, in the present study, 79.9% affirmed that they brushed their teeth twice or more a day. The differences in this aspect may be related to the differences in the oral hygiene habits typical of the country of origin of the study. Culturally, in Spain, especially among younger women, tooth brushing is generally widespread, as can be seen in the data of the last national epidemiological study, in which it was found that 23.6% of adults reported brushing their teeth at least once a day, and more than 65% did it two or more times a day [19].

In El-Mahdi and Mudawi’s study [30] (Sudan, 2016), 66% of the women had low levels of oral self-care, while in the present study, a better level of self-care was observed among pregnant women (21.6% low, 67.6% medium, 10.8% high). In this same investigation, the results related to the state of oral health referred by the pregnant women coincide with the present study, since the medium oral health state prevailed, followed by the low, and finally the high. In the study by Martínez-Beneyto et al. [24], the level of perceived oral health was grouped into excellent, very good, good, and poor, with a predominance of the good level (64.7%), comparable to the medium level of our study and therefore, there is also a coincidence with the present study. On the other hand, in the study by Gaszynska et al. [31] (Poland, 2015), the state of oral health prevailed as medium (59.6%), followed by good (25%), and finally 14.7% considered it bad. However, this does not coincide with the studies by Aguilar-Cordero et al. [25], Avula et al. [27], and Bogges et al. [32] (United States, 2010), where a predominance of the good level was observed.

In the study by Keirse and Plutzer [33] (Australia, 2010), a significant association between the referred oral health state among pregnant women and the level of self-care was found. However, in the present study, the said factors were not related significatively (*p* = 0.222).

A study carried out in New York by Baker et al., in 2016 [34], found that women with better hygienic practices and oral health had better knowledge about oral health. This coincides with our study, in which a significant association between general knowledge and self-care (*p* = 0.037) was found. Likewise, a significant association between referred oral health state among pregnant women and general knowledge about oral health (*p* = 0.012) was found. These results also match with the investigations carried out by Gaszynska et al. [31] and Boggess et al. [32]. The same happens between referred oral health state and level of education among pregnant women, as shown in other investigations [24,27,29].

In the present study, a significant association between the referred oral health state, nationality, level of education, and the possession of a paid job was also found. It should be noted that the majority of women that had a paid job were Spanish, which could act as a confounding factor, and in fact it was not significant in the logistic regression analysis. However, it should be highlighted that both Spanish and foreign women presented a similar level of education.

Regarding the level of knowledge about oral health, in the study by Aguilar-Cordero et al. [25], the predominant level of knowledge was the medium (64%), followed by the low level (26%), while the high represented only 10%. Although the percentages differ with the present study, the distribution is similar, since the highest percentage of general knowledge and knowledge in prevention are also represented by the medium levels of knowledge (55.4% and 66.9%, respectively). This also coincides with other available literature [30,35,36,37]. However, there are also studies in which the prevalent levels of general knowledge were low or very low [22,38].

As in the present study, other studies have also evaluated the level of knowledge in prevention regardless of the level of general knowledge in oral health [22,37,38]. In the study conducted by Núñez et al. in Chile in 2013 [37], it was found that 78.9% of the women had high knowledge related to prevention in oral health, and 64% in the study by Sotomayor et al. [38] conducted in Peru in 2012, while only 23.7% of the present study’s sample showed high levels of knowledge in oral health prevention. However, our results are similar, in terms of the percentage of pregnant women with a medium level of knowledge in prevention, to those reported by Barrios in Peru in 2012 [22].

In two case-control studies conducted in Iran [39,40], it was studied how an educational talk on oral health influences the level of knowledge of pregnant women. It was seen that the group receiving training had higher levels of knowledge. Similarly, in a study conducted in North Carolina (USA) by Bogges et al. [32], there was a statistically significant association between attending a prevention and oral health talk with the level of knowledge. Likewise, in the present study, a quasi-significant association was obtained between attending an oral health education workshop and the level of knowledge in prevention (*p* = 0.051), despite the fact that only 10% of pregnant women had attended one of the training workshops on oral health care that were offered at the Health Center. George et al. also reported the low participation in training proposals offered to pregnant women on oral health care [41], which highlights the greater importance that should be given to training in preventive care and oral health, both for the pregnant women and for the baby.

In the present study, it has been confirmed that women over 30 years of age have higher levels of knowledge than those between 20 and 30 years old (*p* = 0.029), a fact that coincides with the results reported by other authors [25,30,35,37]. However, in the study of Barrios et al., conducted in Peru [22], it was found that the age group of 21–30 years represented the group with the highest level of knowledge in oral health.

According to the studies by Barbieri et al. [35] and Núñez et al. [37], pregnant women who already had children presented better knowledge than those who were in their first pregnancy. In the results of the present investigation, however, a better level of general knowledge in oral health was found in primiparous women. In this sense, it should be noted that there was a significantly higher percentage of primiparous women among Spanish women than among foreign women (*p* = 0.032), so the number of pregnancies could be a confounding factor, since it would be nationality (Spanish or foreign) which would determine the best level of knowledge. In addition, in the study by Baker et al. [34] conducted in the United States, the previous birth of a child did not significantly affect the levels of knowledge.

In the study by Aguilar-Cordero et al. [25], as the trimester of pregnancy increased, the percentage of good oral knowledge increased. This does not coincide with the results found in the present study, since no association or a statistically significant trend was found between the gestation trimester and the level of knowledge. On the other hand, in the study by Barrios conducted in Peru [22], a significant association was found between the trimester and the level of knowledge. Women who were in the second trimester were the ones that represented the highest percentage within the group of good knowledge, followed by the first trimester, and finally the third.

In the present study, a statistically significant association between the level of general knowledge about oral health and level of education was found, which matches with available literature [22,27,29,30,32,35,37,42,43].

Overall, coincident results can be seen between previous researches carried out in the field; highlighting that level of education, nationality, self-care, and knowledge on prevention and oral health are the predominant factors influencing in the level of general knowledge on oral health among pregnant women. Providing a systematic and updated assessment of the current state in this field is crucial to allow for an evaluation of the actions taken to encourage an improved oral health in the community. The fact that the results from this study match those from previous research is a definite indicator of the lack of development in the promotion of oral health among pregnant women or an insufficiency of the current measures established for this purpose.

Considering the results obtained, it would be desirable to implement training activities, on self-care in oral health and care of the baby’s mouth. These activities should be promoted by all health personnel who are involved in the control of the pregnant woman’s health, since it they in whom she places her trust during this stage of many physiological, metabolic, and psychological changes.

## 5. Conclusions

According to the results obtained, it can be affirmed that the educational level, the nationality, the level of self-care, and knowledge on prevention in oral health were the factors that determined a better level of general knowledge about oral health among pregnant women.

## Figures and Tables

**Table 1:** Codification used to group the level of self-care, referred oral health state, basic knowledge about preventive care of the infant’s mouth, and general knowledge about oral health.

Variable	Indicator	Value
Oral self-care	5 questions II-1 to II-5	Low (0–2 points) = 0
		Medium (3–4 points) = 1
		High (5 points) = 2
Referred oral health state	3 questions II-6 to II-8	Low (0–1 points) =0
		Medium (2 points) = 1
		High (3 points) = 2
Knowledge about prevention in oral health	9 questions III-1 to III-9	Low (0–3 points) = 0
		Medium (4–6 points) = 1
		High (7–9 points) = 2
General knowledge about the oral cavity	17 questions III-10 to III-26	Low (0–6 points) = 0
		Medium (7–12 points) = 1
		High (13–17 points) = 2

**Table 2:** General descriptive data of the sample.

			Freq.	Perc.
Nationality	Spanish		112	80.6%
	Foreign		27	19.4%
Time living in Spain (for the foreigners)	Up to 5 years		9	33.33%
	>5 years		18	66.6%
Paid work	Yes		79	56.8%
	No		60	43.2%
Type of job	Technicians, scientific, and intellectual professionals		34	44.15%
	Technicians: support professionals.		8	10.39%
	Accountants, administration, and other office jobs.		12	15.59%
	Catering, personal, protection, and sales service workers.		23	29.87%
Level of education	Primary		22	15.8%
	Secondary		80	57.6%
	University		37	26.6%
First pregnancy	Yes		79	56.8%
	No.	1 child	42	30.2%
		2 children	11	7.9%
		3 children	7	5.0%
Pregnancy trimester	1		41	29.5%
	2		41	29.5%
	3		57	41.0%
Assistance to talks about oral health education	Yes		14	10.1%
	No		125	89.9%

**Table 3:** Oral hygiene and self-care.

		Freq.	Perc.	Score *
Tooth brushing frequency	<once a day	1	0.7%	0
	Once a day	27	19.4%	1
	>once a day	111	79.9%	1
Use of fluoridated toothpaste	Yes	79	56.8%	1
	No	27	19.4%	0
	Does not know	33	23.7%	0
Use of dental floss or interdental brush	Yes	57	41.0%	1
	No	82	59.0%	0
Lingual brushing	Yes	85	61.2%	1
	No	54	38.8%	0
Last visit to the dentist	1 year	33	23.7%	1
	>1 year	47	33.8%	0
	<1 year	59	42.4%	1

* Score used for the codification.

**Table 4:** Referred oral health state among pregnant women.

		Freq.	Perc.	Score *
Presence of caries	Yes.	37	26.6%	0
	Does not know.	43	30.95%	0
	No. All of them are treated.	45	32%	1
	Has never had caries.	14	10.1%	1
Gum bleeding	Yes, spontaneously.	22	15.8%	0
	Yes, while brushing.	47	33.8%	0
	Only during pregnancy.	40	28.8%	1
	Never.	30	21.6%	1
Oral pain or infection in the last 10 months.	Yes.	37	26.6%	0
	No.	102	73.4%	1

* Score used for the codification.

**Table 5:** Knowledge about prevention in oral health among pregnant women.

	Aware 1 Point *		Not Aware 0 Points *	
	Freq.	Perc.	Freq.	Perc.
Preventive measures in oral health.	26	18.7%	113	81.3%
Usefulness of tooth brushing.	100	71.9%	39	28.1%
Usefulness of fluoridated for teeth.	119	85.6%	20	14.4%
Importance of cleaning the child’s mouth before teeth eruption.	86	61.9%	53	38.1%
Importance of cleaning the child’s mouth after teeth eruption.	117	84.2%	22	15.8%
Harmful effect of sleeping with a pacifier or bottle soaked in sweet substance.	131	94.2%	8	5.8%
Not offering night breastfeeding on demand for the baby after teeth eruption.	21	15.1%	118	84.9%
If breastfeeding on demand, fluoridated paste may decrease the risk of tooth decay.	50	36%	89	64%
Harmful effect of finger sucking or the use of pacifier beyond 2 years old.	116	83.5%	23	16.5%

* Score used for the codification.

**Table 6:** General knowledge about different aspects of the mother’s and baby’s oral health.

	Aware 1 Points *		Not Aware 0 Points *	
	Freq.	Perc.	Freq.	Perc.
Effect of the mother’s diet on the formation of the fetus’s teeth	52	37.4%	87	62.6%
When to take the baby to the dentist for the first time	28	20.1%	111	79.9%
What is bacterial plaque	100	71.9%	39	28.1%
Early childhood caries	51	36.7%	88	63.3%
What is gingivitis	104	74.8%	35	25.2%
What is periodontal disease	51	36.7%	88	63.3%
Moment of greater risk of caries in women’s lives	66	47.5%	73	52.5%
Dental radiography with adequate protection during pregnancy	16	11.5%	123	88.5%
Use of anesthesia during pregnancy	23	16.5%	116	83.5%
Dietary and lifestyle habits affect the health of children	128	92.1%	11	7.9%
Health problems of the pregnant woman condition the baby’s health	54	38.8%	85	61.2%
Where does calcium for the fetus proceed from	96	69.1%	43	30.9%
When do temporary teeth start to form	10	7.2%	129	92.8%
Age of eruption of temporary teeth	84	60.4%	55	39.6%
How many temporary teeth are there	19	13.7%	120	86.3%
When to start cleaning the baby’s mouth	35	25.2%	104	74.8%
Fluoride reinforces the enamel of the baby’s tooth	39	28.1%	100	71.9%

* Score used for the codification.

**Table 7:** Assessment of level of self-care, oral health state, and knowledge.

	Low		Medium		High	
	Freq.	Perc.	Freq.	Perc.	Freq.	Perc.
Oral hygiene and self-care.	30	21.6%	94	67.6%	15	10.8%
Referred oral health state.	58	41.7%	52	37.4%	29	20.9%
Knowledge of prevention in oral health.	13	9.4%	93	66.9%	33	23.7%
General knowledge about oral health.	62	44.6%	77	55.4%	0.0	0.0%

**Table 8:** Association between age and the five blocks of the questionnaire.

	Age in Years			*p*
	Low	Medium	High	
Oral self-care and hygiene.	30.03 ± 5.7	31.85 ± 5.35	31.53 ± 5.29	0.281
Referred oral health state.	30.03 ± 5.83 *	31.52 ± 4.77	34.03 ± 4.77 *	0.009
Knowledge about prevention in oral health.	29.92 ± 5.33	31.82 ± 5.82	30.91 ± 4.18	0.415
General knowledge about oral health.	30.31 ± 6 *	32.32 ± 4.78 *	-	0.029

Values with * are significantly different.

**Table 9:** Association between the different variables.

		Self-Care				Referred Oral Health State				Knowledge on Prevention				General Knowledge		
		Low	Medium	High	*p*	Low	Medium	High	*p*	Low	Medium	High	*p*	Low	Medium	*p*
Nationality	Spanish	19.6%	72.3%	8%	0.031	35.7%	44.1%	23.2%	0.014	8.9%	64.3%	26.8%	0.228	39.3%	60.7%	0.010
	Foreigner	29.6%	48.1%	22.2%		66.7%	22.2%	11.1%		11.1%	77.8%	11.1%		66.7%	33.3%	
Paid job	No	27.4%	62.9%	9.7%	0.322	50%	35.5%	14.5%	0.128	11.3%	64.5%	24.2%	0.758	58.1%	41.4%	0.004
	Yes	16.9%	71.4%	11.7%		35.1%	39%	20.6%		7.8%	68.8%	23.4%		33.8%	66.2%	
Type of job	Housewife	27.4%	62.9%	9.7%	0.701	50%	35.5%	14.5%	0.002	11.3%	64.5%	24.2%	0.178	58.1%	41.9%	0.068
	Technicians, scientific, and intellectual professionals	13.3%	73.5%	14.7%		14.7%	52.9%	32.4%		0.0%	73.5%	26.5%		32.4%	67.6%	
	Technicians: support professionals.	6.7%	62.5%	12.5%		12.5%	50%	37.5%		25.0%	37.5%	37.5%		25.0%	75.0%	
	Accountants. Administration, and other office jobs.	16.7%	83.3%	0.0%		58.3%	8.3%	33.3%		0.0%	83.3%	16.7%		33.3%	66.7%	
	Catering, personal, protection, and sales service workers.	21.7%	65.2%	13.0%		60.9%	30.4%	8.7%		17.4%	65.2%	17.4%		39.1%	60.9%	
Education	Primary	40.9%	50.0%	9.1%	0.055	50%	50%	0.0%	0.030	4.5%	63.6%	31.8%	0.495	68.2%	31.8%	0.015
	Secondary/University	17.9%	70.9%	11.1%		40.2%	35%	24.8%		10.3%	67.5%	22.2%		40.2%	59.8%	
First pregnancy	Yes	17.7%	68.4%	13.9%	0.230	39.2%	39.2%	21.5%	0.789	11.4%	67.1%	21.5%	0.550	36.7%	63.3%	0.032
	No	26.7%	66.7%	6.7%		45%	35%	20%		6.7%	66.7%	26.7%		55.0%	45.0%	
Pregnancy trimester	1	17.1%	73.2%	9.8%	0.055	46.3%	34.1%	19.5%	0.855	4.9%	26.8%	26.8%	0.514	51.2%	48.8%	0.403
	2	22.0%	61.0%	17.1%		41.5%	41.5%	17.1%		14.6%	17.1%	17.1%		36.6%	63.4%	
	3	24.6%	68.4%	7.0%		38.6%	36.8%	24.6%		8.8%	26.3%	26.3%		45.6%	54.4%	
Assistance to talks about oral health promotion	Yes	21.4%	71.4%	7.1%	0.893	35.7%	42.9%	21.4%	0.878	7.1%	42.9%	50.0%	0.051	50.0%	50.0%	0.668
	No	21.6%	67.2%	11.2%		42.4%	36.8%	20.8%		9.6%	69.6%	20.8%		44.0%	56.0%	
Self-care	Low					53.3%	30%	16.7%	0.302	3.3%	80%	16.7%	0.251	60%	40%	0.037
	Medium					41.5%	38.3%	20.2%		11.7%	64.9%	23.4%		43.6%	56.4%	
	High					20%	46.7%	33.3%		6.7%	53.3%	40%		20%	80%	
Referred oral health state	Low	29.1%	65.5%	5.5%	0.222					10.9%	69.1%	20%	0.925	58.6%	41.4	0.012
	Medium	15.9%	71.0%	13.0%						8.7%	65.2%	26.1%		38.5%	61.5	
	High	20.0%	60.0%	20.0%						6.7%	66.7%	26.7%		27.6%	72.4	
Knowledge on prevention	Low	7.7%	84.6%	7.7%	0.251	53.8%	38.5%	7.7%	0.394					69.2%	30.8	0.003
	Medium	25.8%	65.6%	8.6%		43%	33.3%	23.7%						49.5%	50.5	
	High	15.2%	66.7%	18.2%		33.3%	48.5%	18.2%						21.%	78.8	

**Table 10:** Factors related to the general knowledge about oral health among pregnant women.

	B	E. Standard	Wald	Sig.	Exp(B)	95% C.I. for EXP(B)	
						Inferior	Superior
Nationality (Spanish/foreigner)	1.280	0.538	5.652	0.017	3.596	1.252	10.326
Education (primary/secondary or university)	−1.470	0.569	6.678	0.010	0.230	0.075	0.701
Self-care_low			5.169	0.075			
Self-care_medium	−1.924	0.850	5.124	0.024	0.146	0.028	0.772
Self-care_high	−1.572	0.788	3.975	0.046	0.208	0.044	0.974
Knowledge_prevention_low			9.509	0.009			
Knowledge_prevention_medium	−2.391	0.812	8.673	0.003	0.092	0.019	0.449
Knowledge_prevention_high	−1.273	0.534	5.679	0.017	0.280	0.098	0.798
Constant	2.028	0.908	4.985	0.026	7.597		

## References

[1] Hartnett E., Haber J., Krainovich-Miller B., Bella A., Vasilyeva A., Lange Kessler J. (2016). Oral Health in Pregnancy. J. Obstet. Gynecol. Neonatal Nurs..

[2] Petersen P.E. (2008). World Health Organization global policy for improvement of oral health—World Health Assembly 2007. Int. Dent. J..

[3] González-Jaranay M., Téllez L., Roa-López A., Gómez-Moreno G., Moreu G. (2017). Periodontal status during pregnancy and postpartum. PLoS ONE.

[4] Guggenheimer J., Moore P.A. (2003). Xerostomía: Etiology, recognition and treatment. J. Am. Dent. Assoc..

[5] Silk H., Douglass A.B., Douglass J.M., Silk L. (2008). Oral health during pregnancy. Am. Fam. Physician.

[6] Wu M., Chen S.W., Jiang S.Y. (2015). Relationship between Gingival Inflammation and Pregnancy. Mediat. Inflamm..

[7] Bilińska M., Sokalski J. (2016). Pregnancy gingivitis and tumor gravidarum. Ginekol. Pol..

[8] Mumghamba E., Manji K., Michael J. (2006). Oral hygiene practices, periodontal conditions, dentition status and self-reported bad mouth breath among young mothers, Tanzania. Int. J. Dent. Hyg..

[9] Steinberg B.J., Hilton I.V., Iida H., Samelson R. (2013). Oral Health and Dental Care during Pregnancy. Dent. Clin. N. Am..

[10] Teshome A., Yitayeh A. (2016). Relationship between periodontal disease and preterm low birth weight: Systematic review. Pan Afr. Med. J..

[11] Sanz M., Kornman K., Working group 3 of the joint EFP/AAP workshop (2013). Periodontitis and adverse pregnancy outcomes: Consensus report of the Joint EFP/AAP Workshop on Periodontitis and Systemic Diseases. J. Clin. Periodontol..

[12] Finlayson T.L., Gupta A., Ramos-Gomez F.J. (2017). Prenatal Maternal Factors, Intergenerational Transmission of Disease, and Child Oral Health Outcomes. Dent. Clin. N. Am..

[13] Berkowitz R.J. (2006). Mutans streptococci: Acquisition and transmission. Pediatr. Dent..

[14] Tanzer J.M., Livingston J., Thompson A.M. (2001). The microbiology of primary dental caries in humans. J. Dent. Educ..

[15] Dzidic M., Collado M.C., Abrahamsson T., Artacho A., Stensson M., Jenmalm M., Mira A. (2018). Oral microbiome development during childhood: An ecological succession influenced by postnatal factors and associated with tooth decay. ISME J..

[16] Abou E.l., Fadl R., Blair M., Hassounah S. (2016). Integrating Maternal and Children’s Oral Health Promotion into Nursing and Midwifery Practice—A Systematic Review. PLoS ONE.

[17] Lucey S.M. (2009). Oral health promotion initiated during pregnancy successful in reducing early childhood caries. Evid. Based Dent..

[18] Deshraj J., Nishant A., Anshu G., Sandhya J. (2016). Dental Health Care in Pregnancy: A Survey and Literature Review. Int. J. Sci. Res..

[19] Bravo Pérez M., Almerich Silla J.M., Ausina Márquez V., Avilés Gutiérrez P., Blanco González J.M., Canorea Díaz E. (2016). Oral health survey in Spain 2015. RCOE.

[20] Harris R.V., Nicoll A.D., Adair P.M., Pine C. (2004). Risk factors for dental caries in young children: A systematic review of the literature. Community Dent. Health.

[21] ACFF Alliance for a Cavity Free Future. http://family.allianceforacavityfreefuture.org/en/us/our-goals.

[22] Barrios Lambruschini D. (2012). Level of Knowledge about Oral Health in Pregnant Women of HONADOMANI “San Bartolomé”. Ph.D. Thesis in dental surgeon.

[23] ISCO-88 International Classification of Occupations. https://ec.europa.eu/eurostat/documents/1012329/6070763/ISCO88.pdf/192120ae-49cb-4f24-bfbc-06f054471e3b.

[24] Martínez-Beneyto Y., Vera-Delgado M.V., Pérez L., Maurandi A. (2011). Self-reported oral health and hygiene habits, dental decay, and periodontal condition among pregnant European women. Int. J. Gynaecol. Obstet..

[25] Aguilar-Cordero M.J., Rivero-Blanco T., Lasserrot-Cuadrado A., Núñez-Negrillo M., Gil-Montoya J.A., Sánchez-López A.M. (2018). Level of knowledge about oral health of pregnant patients: A descriptive study. JONNPR.

[26] Assery M.K. (2016). A 22 year comparison survey of dental knowledge in Al-Jubail antenatal clinic population. Saudi Dent. J..

[27] Avula H., Mishra A., Arora N., Avula J. (2013). KAP assessment of oral health and adverse pregnancy outcomes among pregnant women in Hyderabad, India. Oral Health Prev. Dent..

[28] Gaffar B.O., El Tantawi M., Al-Ansari A., Al-Agl A.S. (2016). Association between oral health knowledge and practices of Saudi pregnant women in Dammam, Saudi Arabia. East. Mediterr. Health J..

[29] Bamanikar S., Kee L.K. (2013). Knowledge, attitude and practice of oral and dental healthcare in pregnant women. Oman Med. J..

[30] El-Mahdi I., Mudawi G. (2016). Oral health status, knowledge and practice among pregnant women attending Omdurman maternity hospital, Sudan. East. Mediterr. Health J..

[31] Gaszyńska E., Klepacz-Szewczyk J., Trafalska E., Garus-Pakowska A., Szatko F. (2015). Dental awareness and oral health of pregnant women in Poland. Int. J. Occup. Med. Environ. Health.

[32] Boggess K.A., Urlaub D.M., Moos M.K., Polinkovsky M., El-Khorazaty J., Lorenz C. (2011). Knowledge and beliefs regarding oral health among pregnant women. J. Am. Dent. Assoc..

[33] Keirse M.J., Plutzer K. (2010). Women’s attitudes to and perceptions of oral health and dental care during pregnancy. J. Perinat. Med..

[34] Baker S.D., Quiñonez R.B., Boggess K., Phillips C. (2016). Pregnant Women’s Infant Oral Health Knowledge and Beliefs: Influence of Having Given Birth and of having a Child in the Home. Matern. Child Health J..

[35] Barbieri W., Verzinhasse Peres S., Britto Pereira C., Peres Neto J., Luz Rosário de Sousa M., Cortellazzi K.L. (2018). Sociodemographic factors associated with pregnant women’s level of knowledge about oral health. Einstein.

[36] Toscano-García I., Luengo-Fereir J.A., Anaya-Álvarez M., Carlos-Medrano L.E., López-Ávila L.G., Márquez-Sánchez S.S. (2016). Evaluation of knowledge level in oral health in pregnant women attending the Women’s Hospital, Zacatecas – México. Multidiscip. Health Res..

[37] Núñez J., Moya P., Monsalves M.J., Landaeta S. (2013). Oral Health Level of Knowledge and Use of Dental GES in Puerperal Patients at a Private Clinic, Santiago, Chile. Int. J. Odontostomat..

[38] Sotomayor Camayo J., Reyes Soto S., Ochoa Ttaje J., Malima Medina A., Correa Olaya E., Arieta Miranda J., Silva Valencia M., Watanabe Velásquez R., Ayala de la Vega G., Chuquihuaccha Granda V. (2012). Level of knowledge in prevention of oral health in pregnant women treated in two national peruvian hospitals. Odontol. Sanmarquina..

[39] Ghaffari M., Rakhshanderou S., Safari-Moradabadi A., Torabi S. (2018). Oral and dental health care during pregnancy: Evaluating a theory-driven intervention. Oral Dis..

[40] Bahri N., Tohidinik H.R., Bahri N., Iliati H.R., Moshki M., Darabi F. (2015). Educational intervention to improve oral health beliefs and behaviors during pregnancy: A randomized-controlled trial. J. Egypt. Public Health Assoc..

[41] George A., Johnson M., Blinkhorn A., Ajwani S., Bhole S., Yeo A.E., Ellis S. (2013). The oral health status, practices and knowledge of pregnant women in south-western Sydney. Aust. Dent. J..

[42] Hom J.M., Lee J.Y., Divaris K., Baker A.D., Vann W.F. (2012). Oral health literacy and knowledge among patients who are pregnant for the first time. J. Am. Dent. Assoc..

[43] Hashim R. (2012). Self-reported oral health, oral hygiene habits and dental service utilization among pregnant women in United Arab Emirates. Int. J. Dent. Hyg..

